# Bombesin family receptor and ligand gene expression in human colorectal cancer and normal mucosa

**DOI:** 10.1054/bjoc.1998.0888

**Published:** 1999-12-08

**Authors:** H S Chave, A C Gough, K Palmer, S R Preston, J N Primrose

**Affiliations:** University Surgical Unit, Southampton General Hospital, Tremona Road, Southampton, S09 4XY, UK; Academic Surgical Unit, St James's University Hospital, Leeds, UK

**Keywords:** colorectal cancer, bombesin, gastrin releasing peptide, neuromedin B, reverse transcription polymerase chain reaction, in situ hybridization

## Abstract

Bombesin-like peptides and their receptors are widely distributed throughout the gut and are potential mitogens for a number of gastrointestinal (GI) cancers. We have analysed the expression of bombesin-like peptides and their receptor subtypes in normal and neoplastic colorectal tissue. Expression was analysed by reverse transcription polymerase chain reaction (RT-PCR) using receptor and ligand subtype-specific primers and then expression localized by in situ hybridization (ISH) with riboprobes synthesized by in vitro transcription of cloned PCR products. Colorectal cancer tissue and matched normal mucosa from 23 patients were studied. Two of these patients had synchronous adenomatous polyps and two had synchronous hepatic metastases which were also studied. An additional two patients with adenomatous polyps were studied along with matched normal mucosa. Gastrin releasing peptide (GRP) receptor and ligand expression was present in all samples but with overall greater expression in the tumour samples. Neuromedin B (NMB) receptor expression was not detectable. NMB ligand was detected in all but one mucosal sample with overall overexpression in the tumour samples. Bombesin receptor subtype 3 (BRS-3) receptor expression was not detectable. These data support the possibility that GRP may be an autocrine growth factor in colorectal cancer. © 2000 Cancer Research Campaign


*British Journal of Cancer* (2000) **82**(1), 124–130
© 2000 Cancer Research Campaign
Article no. bjoc.1999.0888

### Bombesin family receptor and ligand gene expression in human colorectal cancer and normal mucosa

**HS Chave[1], AC Gough[1], K Palmer[1], SR Preston[2] and JN Primrose[1]**

[1]University Surgical Unit, Southampton General Hospital, Tremona Road, Southampton S09 4XY, UK; [2]Academic Surgical Unit, St James's University Hospital, Leeds, UK

**Summary** Bombesin-like peptides and their receptors are widely distributed throughout the gut and are potential mitogens for a number of gastrointestinal (GI) cancers. We have analysed the expression of bombesin-like peptides and their receptor subtypes in normal and neoplastic colorectal tissue. Expression was analysed by reverse transcription polymerase chain reaction (RT-PCR) using receptor and ligand subtype-specific primers and then expression localized by in situ hybridization (ISH) with riboprobes synthesized by in vitro transcription of cloned PCR products. Colorectal cancer tissue and matched normal mucosa from 23 patients were studied. Two of these patients had synchronous adenomatous polyps and two had synchronous hepatic metastases which were also studied. An additional two patients with adenomatous polyps were studied along with matched normal mucosa. Gastrin releasing peptide (GRP) receptor and ligand expression was present in all samples but with overall greater expression in the tumour samples. Neuromedin B (NMB) receptor expression was not detectable. NMB ligand was detected in all but one mucosal sample with overall overexpression in the tumour samples. Bombesin receptor subtype 3 (BRS-3) receptor expression was not detectable. These data support the possibility that GRP may be an autocrine growth factor in colorectal cancer. © 2000 Cancer Research Campaign

**Keywords:** colorectal cancer; bombesin; gastrin releasing peptide; neuromedin B; reverse transcription polymerase chain reaction; in situ hybridization

Colorectal carcinoma is a significant cause of morbidity and mortality world-wide. In the UK, it is the second most common cause of cancer death, accounting for 19 000 deaths per year (Cancer Research Campaign Factsheets, 1993). Although understanding of the molecular basis of colorectal carcinoma is rapidly increasing (Groden et al, 1991; Leache et al, 1993), many additional factors remain to be elucidated. It is clear that genetic factors are important in colorectal tumorigenesis (Cannon-Albright et al, 1988; Vogelstein et al, 1988) but it is likely that hormones and locally acting growth factors are also important in the development and progression of the disease. Breast cancer is a paradigm in this respect, exhibiting both genetic alterations and sensitivity to endocrine factors, which has been exploited therapeutically with the use of oestrogen receptor antagonists (Santen et al, 1990). The gastrointestinal tract is a rich source of peptide hormones. Functional conservation of peptide hormone receptors following malignant transformation in the colon may permit susceptibility to the therapeutic manipulation of the actions of endogenous trophic hormones.

Bombesin, an amphibian tetradecapeptide, exerts a wide range of effects on the mammalian gastrointestinal tract and bombesin-like immunoreactivity has been demonstrated in the submucosal and myenteric plexuses throughout the gut. The search for mammalian bombesin-like peptides resulted in the discovery of gastrin releasing peptide (GRP) (McDonald et al, 1979) and neuromedin B (NMB) (Minamino et al, 1983). These bombesin-like peptides regulate gut motility, influence secretion of enteric peptide hormones and pancreatic exocrine secretion (reviewed in Sunday et al, 1988). The genes encoding GRP and NMB have been cloned and sequenced. A single GRP-encoding gene has been localized to chromosome 18 (Spindel et al, 1984; Naylor et al, 1987). Alternate splicing produces three different transcribed GRP mRNAs which are translated into GRP preprohormones each differing in their C-terminal extension peptide (Sausville et al, 1986; Spindel et al, 1986). Similarly, molecular cloning from human hypothalamus has identified a single NMB gene on chromosome 15, two transcribed mRNAs and a 76 amino acid NMB preprohormone (Krane et al, 1988). The mechanism responsible for the production of two NMB mRNAs has yet to be determined. The natural ligand for BRS-3 has not been identified, but studies with a synthetic antagonist have demonstrated high affinity binding to the BRS-3 receptor (Mantey et al, 1997).

To date three bombesin-like peptide receptors have been isolated and cloned. The GRP receptor is found in the gut, from oesophagus to rectum (Sunday et al, 1988; reviewed in Spindel et al, 1993); the NMB receptor is present in the oesophageal muscularis mucosa (von Schrenck et al, 1989) and smooth muscle cells from the rectosigmoid colon (Bitar et al, 1993); and expression of the bombesin receptor subtype 3 (BRS-3) is limited, in rats, to germ cells in the testes but is present in a range of human lung cancer cells (Corjay et al, 1991; Fathi et al, 1993). The human GRP receptor and NMB receptor were cloned from placental and peripheral blood genomic libraries, and human small-cell lung cancer (SCLC) cell lines (Corjay et al, 1991). The GRP receptor gene was localized to the X chromosome on the human genome between p11 and q11 (Schantz et al, 1991) and the NMB receptor

*Received 7 December 1998*
*Revised 22 June 1999*
*Accepted 7 July 1999*

*Correspondence to:* JN Primrose gene to chromosome 6 q21 on the human genome (Daly et al, 1991). Later, the BRS-3 gene was mapped on human chromosome X (Fathi et al, 1993).

Bombesin-like peptides are mitogenic for Swiss 3T3 fibroblasts (Rozengurt et al, 1983) and human SCLC cell lines (Weber et al, 1985). In vivo and in vitro studies have demonstrated mitogenic effects on a number of gastrointestinal mucosal and cancer cells (Preston et al, 1996). In SCLC and human pancreatic cancer cells an autocrine loop has been demonstrated (Cuttita et al, 1985; Wang et al, 1996).

We have previously shown, by means of radioligand binding studies, that GRP receptors are expressed in 24% of colorectal cancers (Preston et al, 1995), which is in agreement with previous reports (Radulovic et al, 1992). These binding sites are not detected in normal colonic mucosa. High-affinity functional GRP binding sites are present on the human cancer cell line Colo320HSR (Hirai et al, 1993; Preston et al, 1994). Inhibition of the growth of Colo320HSR by a GRP antagonist has also been demonstrated in vivo using a clonogenic assay (Preston at al, 1994). In this cell line at least, GRP may act as an autocrine growth factor.

The possibility that GRP is an autocrine growth factor in colorectal cancer warrants further investigation using more powerful methodologies. In the absence of validated antibodies for the GRP receptor, we have used the molecular techniques of reverse transcription polymerase chain reaction (RT-PCR) and in situ hybridization (ISH) to detect and then localize steady-state levels of mRNA for the bombesin-like peptides and their receptors in human colorectal cancers and matched normal mucosa.

### MATERIALS AND METHODS

Twenty-three specimens of colorectal cancer tissue and paired normal mucosal samples, four polyps and two colorectal liver metastases were studied (Table 1).

### Tissue collection

All samples were obtained fresh, snap-frozen in liquid nitrogen and stored at –80°C.

### RNA extraction

Total cellular RNA was extracted from frozen tissue using the RNeasy kit (Qiagen, Dorking, Surrey, UK).

### Reverse transcription

Reverse transcription was performed by 400 U MMLV-RT (Gibco-BRL, Paisley, Scotland) in 1X manufacturers buffer containing 0.5 mM dNTPs, 10 mM dithiothreitol (DTT) and 0.05 units pdT(12–18) primer (Pharmacia, St Albans, Herts, UK). cDNA integrity was verified by PCR amplification of a fragment of the constitutively expressed β-actin gene (primers and conditions obtained from Stratagene).

### Polymerase chain reaction

Primers, determined from published cDNA sequences (Spindel et al, 1984; Krane et al, 1988; Corjay et al, 1991; Fathi et al, 1993) (Table 2), were designed to span an intron hence avoiding confusion between RNA and genomic DNA-derived products. Thermal cycling, with appropriate positive and negative controls, was performed at 0.25 μM final concentration using 250 μM of each dNTP, 1.25 U *Taq* polymerase (Amersham, Little Chalfont, Bucks, UK) in manufacturers buffer containing 2 mM magnesium chloride ($MgCl_2$) and 2% dimethyl sulphoxide (DMSO) (Sigma, Poole, Dorset, UK) for GRP receptor; 2.5 mM $MgCl_2$ without DMSO for GRP ligand; 3 mM $MgCl_2$ and 1% DMSO for NMB receptor; 1.5 mM $MgCl_2$ and 2% DMSO for NMB ligand and BRS-3 receptor. Cycling was carried out on a Techne PHC3 thermal cycler with an initial denaturing step of 3 min at 94°C, followed by 30 cycles of 94°C denaturation for 30 s, annealing at 62°C (GRP-R); 56°C (GRP-L); 56°C (NMB-R); 66°C (NMB-L); 56°C (BRS-3-R) for 30 s and 72°C polymerization for 30 s. This was followed by a final extension step of 72°C for 2 min to ensure completion of all initiated polymerization events.

Products were resolved on 8% non-denaturing polyacrylamide gel, stained with ethidium bromide, visualized by UV transillumination and photographed.

The results of the RT-PCR were semi-quantified by scoring the intensity of the receptor or ligand band compared with the intensity of the β-actin band using the same amount of starting template. By this means we were able to directly compare scores between tumour and mucosa and make a semi-quantatative assessment of their relative abundance. This assessment used a 5 point arbitrary scale; 0 – no expression compared to β-actin; 1–very weak expression; 2 – weak expression; 3 – strong expression; 4 – expression equal to, or greater than, β-actin. For each specimen the PCR was repeated 3 times and the scores added. Thus, a total score range of 0–12 could be obtained. The scoring was performed by an observer unaware of the nature of the products being evaluated.

Confirmation of the nature of the PCR product was accomplished by restriction enzyme digestion and cloning (data not shown). PCR products were cloned into PGEM®-Teasy (Promega), transformed into competent *Escherichia coli* cells and characterized by dideoxy sequencing using the T7 kit obtained from Pharmacia Biotech. For each receptor and ligand subtype cloned, the anticipated PCR product was obtained.

### In situ hybridization

In situ hybridization was performed on a subset of matched tumour–mucosa pairs using a 188 bp digoxigenin labelled riboprobe for GRP-receptor, and 310 bp digoxin-labelled riboprobe for GRP-ligand. Ten micron cryostat sections of frozen tumour were taken and fixed for 4 min in 4% paraformaldehyde at 4°C. After washing twice for 10 min in DEPC-treated water, sections were prehybridized in 40% deionized formamide, 5 × SSPE, 1 × Denhardt's solution, 0.05 M DTT and 1.5 units $ml^{-1}$ sonicated salmon sperm for 1 h at 48°C for GRP-R and 38°C for GRP-L. Hybridization was carried out overnight at the same temperature with 500 μg $ml^{-1}$ of riboprobe in prehybridisation solution with 10% dextran sulphate.

Non-hybridized probe was removed by washing with 2 × SSC (standard saline citrate) for 5 min, 1 × SSC for 5 min and 0.2 × SSC for 10 min. Sections were incubated in blocking solution (3% bovine serum albumin (BSA) in 25 mM Tris-buttered saline (TBS) with 0.1% Triton X) for 30 min at room temperature. Anti-digoxigenin antibody (Boehringer Mannheim, Lewes, Sussex,

© 2000 Cancer Research Campaign

**Table 1** Summary of clinical, pathological and experimental data for the analysis of bombesin-like receptors and ligand mRNA expression in colorectal cancer

| Patient number | Grade (differentiation) | Dukes' stage | Site | *GRP receptor mRNA expression | | *GRP mRNA expression | | *NMB mRNA expression | |
|---|---|---|---|---|---|---|---|---|---|
| | | | | T | N | T | N | T | N |
| 1 | Moderately | A | Rectum | 9 | 5 | 9 | 6 | 8 | 7 |
| 2 | Well | B | Ascending | 3 | 1 | 12 | 4 | 8 | 6 |
| 3 | Moderately | B | Rectum | 3 | 4 | 7 | 3 | 1 | 5 |
| 4 | Moderately | B | Rectum | 9 | 7 | 7 | 5 | 12 | 3 |
| 5 | Moderately | B | Transverse | 6 | 8 | 9 | 11 | 7 | 1 |
| 6 | Poor | B | Ascending | 11 | 5 | 4 | 3 | 12 | 11 |
| 7 | Moderately | C | Ascending | 10 | 9 | 10 | 8 | 8 | 5 |
| 8 | Moderately | C | Descending | 6 | 4 | 7 | 5 | 11 | 5 |
| 9 | Severely dysplastic | TVA polyp | Sigmoid | 6 | 5 | 7 | 6 | 8 | 7 |
| 10 | Moderately | B | Rectum | 8 | 5 | 8 | 8 | 4 | 2 |
| | | TVA polyp | Rectum | 5 | | 5 | | 1 | |
| 11 | Moderately | B | Descending | 10 | 9 | 10 | 6 | 7 | 7 |
| 12 | Moderately | C | Rectum | 8 | 6 | 11 | 9 | 6 | 4 |
| 13 | Moderately | C | Rectum | 8 | 9 | 11 | 11 | 11 | 10 |
| 14 | Poor | D | Rectum | 11 | 9 | 8 | 6 | 7 | 5 |
| | | | Liver met | 8 | | 3 | | 6 | |
| 15 | Well | B | Transverse | 10 | 4 | 12 | 8 | 11 | 12 |
| 16 | Severely dysplastic | TVA polyp | Rectum | 7 | 5 | 7 | 12 | 12 | 4 |
| 17 | Poor | B | Sigmoid | 9 | 4 | 4 | 4 | 4 | 5 |
| 18 | Poor | D | Rectum | 9 | 7 | 4 | 10 | 9 | 5 |
| | | | Liver met | 5 | | 8 | | 5 | |
| 19 | Mod | B | Transverse | 9 | 8 | 10 | 8 | 11 | 2 |
| 20 | Mod | B | Rectum | 5 | 3 | 11 | 9 | 12 | 0 |
| | | TA polyp | Rectum | 2 | | 10 | | 5 | |
| 21 | Mod | B | Sigmoid | 6 | 6 | 4 | 6 | 7 | 6 |
| 22 | Mod | C | Rectum | 9 | 9 | 8 | 6 | 7 | 2 |
| 23 | Poor | C | Rectum | 4 | 8 | 10 | 7 | 12 | 11 |
| 24 | Poor | C | Descending | 5 | 8 | 6 | 4 | 6 | 7 |
| 25 | Well | D | Sigmoid | 10 | 9 | 6 | 8 | 2 | 8 |
| | | | | T>N | 18 | | 17 | | 19 |
| | | | | T=N | 2 | | 3 | | 1 |
| | | | | T<N | 5 | | 5 | | 5 |

T = tumour; N = normal mucosa; TA = tubular adenoma; TVA = tubulovillous adenoma. *Scored as sum of 3 determinations with score 0–4 (i.e. 0–12) when compared to β-actin expression, where 0, no expression; 1, very weak expression; 2, weak expression; 3, strong exprssion; and 4, equally or greater expressed than β-actin.

UK) diluted at 1/5000 in blocking solution was applied and left for 90 min. Unbound antibody was washed off with diluted blocking solution (0.1% in TBS) twice for 5 min. This was followed by incubation with 1/30 dilution of blocking solution twice for 5 min. Hybridized probe was visualized by anti-digoxigenin antibody conjugated to an alkaline phosphatase detection system (Boehringer Mannheim Biochemica). For each sample, a control reaction was carried out on an adjacent tissue section using a sense probe. All prehybridization solutions were guaranteed RNAase free.

### RESULTS

### Pathological details

#### *General*

The pathological details are summarized in Table 1. Tumour, with a sample of normal tissue taken from as far away as possible from the tumour, but not involving the resection margins, was obtained from 14 male and 11 female patients with a median age of 74 years (range 52–86 years).

#### *Cancer*

The nature of all tissues was verified by a consultant histopathologist. All cancers were adenocarcinomas. Three carcinomas were from the ascending colon, three transverse colon, three descending colon, three sigmoid colon and 11 from the rectum. Dukes' stage 'D' has been included to represent all samples of primary tumour where distant metastases were present at the time of the initial resection. Staging of tumours were as follows; 4% Dukes' A, 52% Dukes' B, 31% Dukes' C and 13% Dukes' D. Of tumours, 13% were well differentiated, 65% moderately differentiated and 22% poorly differentiated (Table 1).

The four polyps were all rectal in origin and two were from patients who also had cancers resected. Three were dysplastic villous adenomas and the remaining one a tubular adenoma.

Two liver metastases were taken from patients who had rectal tumours.

 © 2000 Cancer Research Campaign

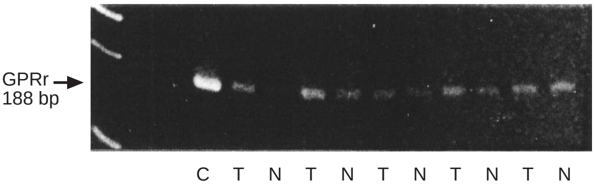

**Figure 1** RT-PCR analysis of GRP receptor mRNA expression in colorectal cancers and matched normal mucosa (all had comparable levels of β-actin mRNA expression)

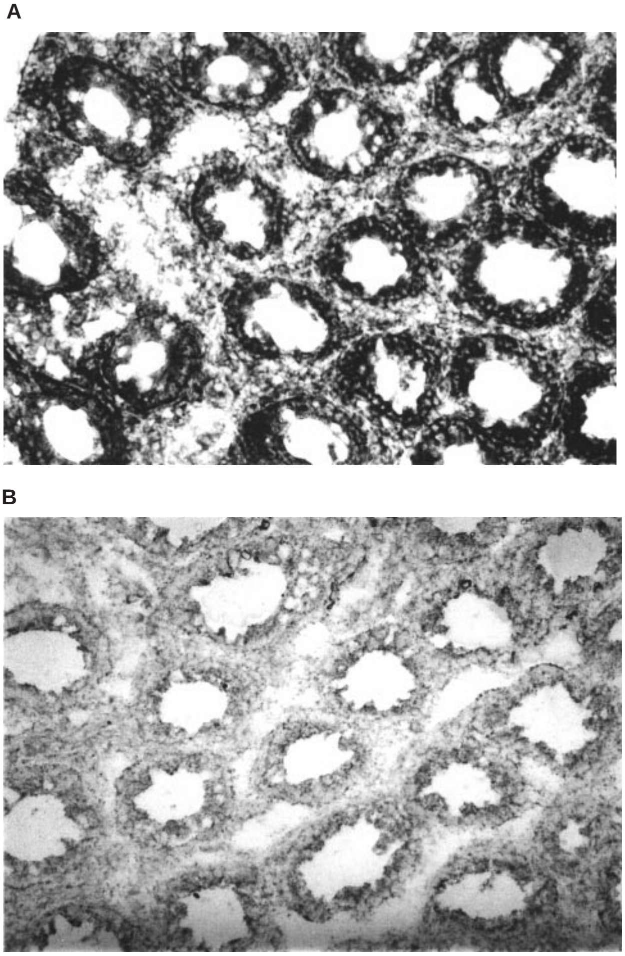

**Figure 2** Frozen sections of normal colonic mucosa following in situ hybridization with antisense (**A**) and sense (**B**) riboprobes specific for GRP receptor (patient 12, Table 1)

### Receptors

All specimens expressed detectable levels of mRNA for the GRP receptor (Table 1). In 16 of the 23 matched cancer–mucosa pairs there was overexpression of GRP receptor mRNA in tumour compared to the mucosa (Figure 1). Two of four polyps studied also demonstrated relative overexpression of GRP receptor mRNA. Relative overexpression in the tumours appeared to be more prevalent in the earlier staged tumours but numbers precluded statistical evaluation. There was no correlation between GRP mRNA expression and degree of differentiation or site of tumour. Both metastases demonstrated lower expression than that of the matched tumour. NMB and BRS-3 receptor mRNA was not detected in any samples.

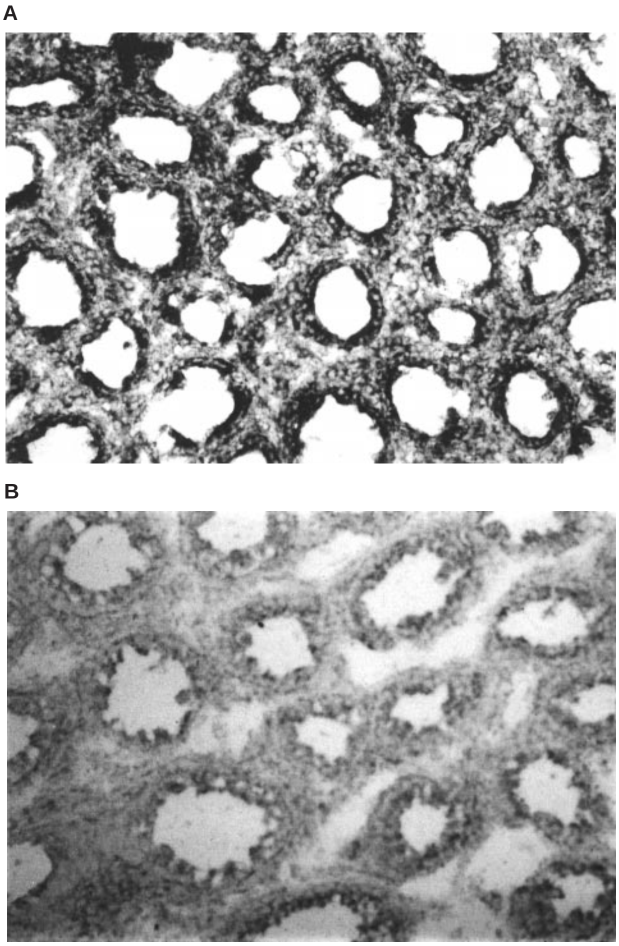

**Figure 3** Frozen sections of normal mucosa following in situ hybridization with antisense (**A**) and sense (**B**) riboprobes specific for GRP ligand

### Ligands

GRP mRNA and the alternate donor site mRNA were identified using primers that flank a potential site for alternate splicing. The products were verified by cloning and sequencing. Both GRP and the alternate donor site mRNA were expressed at detectable levels in all tumour and mucosal samples (Table 1). Comparing tumour to normal mucosa there was apparent overexpression in 16 of the 23 cancer–mucosa pairs. Although there was some variation in the expression of GRP ligand and its spliced variant there was no consistent pattern. One of the four polyps demonstrated greater expression than matched mucosa. One metastasis demonstrated greater expression and the other lower expression than matched tumour.

The NMB-ligand mRNA was expressed in all but one mucosal samples, and in all tumour samples. It was clearly overexpressed in tumour in 17 of the 23 cancer–mucosa pairs (Table 1). Three of the four polyps showed greater expression than matched mucosa. Both metastases showed lower expression than the tumour. Thirteen of the 23 cancer–mucosa pairs showed tumour overexpression of mRNA for GRP receptor, and GRP and NMB ligand.

© 2000 Cancer Research Campaign

A

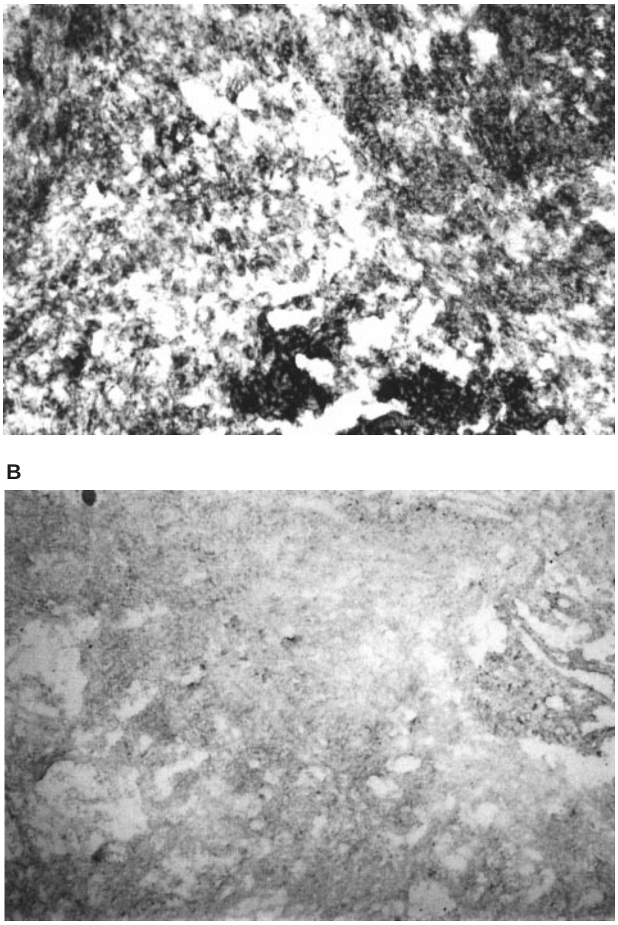

B

**Figure 4** Frozen sections of colorectal cancer following in situ hybridization with antisense (**A**) and sense (**B**) riboprobes specific for GRP receptor

In situ hybridization was used to localize cells expressing mRNA for both the GRP receptor and ligand. All tumour and mucosal samples expressed mRNA for both the receptor and ligand. Expression was localized to the epithelial cells. GRP receptor mRNA expression appeared most prominant at the basolateral aspect of the mucosal cells (Figure 2), whereas GRP ligand mRNA expression appeared to be maximal at the luminal surface (Figure 3). In the tumour samples, although clearly positive for both the receptor and ligand mRNA, no particular pattern of expression could be determined (Figure 4).

### DISCUSSION

In this study we have determined the receptor and ligand expression of the known mammalian bombesin-family peptides in benign and malignant human colon tissue by RT-PCR and ISH. The lack of reliably specific monoclonal antibodies and the relative insensitivity of autoradiography currently renders detection of the gene product problematic. We therefore elected to analyse the steady-state levels of messenger RNA for each receptor and ligand. RT-PCR was chosen because it is an exquisitely sensitive technique that will amplify small quantities of messenger RNA, and in many cases only small amounts of tissue were available for analysis. ISH is less sensitive, but allows nucleic acids to be visualized in their cellular environment and was used to localize mRNA expression in the tissues studied. However, the results of this study must be interpreted with caution because the detection of gene expression, as determined by the presence of mRNA, does not necessarily imply the expression of a functional protein.

**Table 2** RT-PCR primers

| | mRNA | Sequence |
|---|---|---|
| GRP receptor | Sense | GGCGCTCTCGGCAGACAGATAC |
| | Antisense | TAATGAAGGTCTGGTTGGTGC |
| GRP ligand (Abi Olibo) | Sense | ATAGAAGCAAAGGAGAACAG |
| | Antisense | GAAAGATACAGAATAGTCAAA |
| NMB receptor (Oswel) | Sense | TATTTCCTCATACCACTTGC |
| | Antisense | GCTTTCCGTGTTTCCATCTGT |
| NMB ligand (Oswel) | Sense | GGGGGCGCTCGGATGTTCGG |
| | Antisense | TCAGGGAGGTGTGGGGAGCTG |
| BRS-3 receptor | Sense | CTGCGTCTGGATCGTGTCTA |
| | Antisense | TTCGGGATTCAATCTGCTTA |

This study demonstrates for the first time, the presence of the mRNA for GRP and its corresponding receptor and NMB in colorectal cancer and in normal mucosa. Levels of expression are generally higher in tumours. Using ISH we have also localized the expression of the receptor to the epithelium rather than the stroma. Using RT-PCR the GRP receptor appears to be reliably over expressed in at least 70% of colorectal cancers when compared with uninvolved mucosa. Previous radioligand binding studies demonstrated that about 25–40% of cancers express the GRP receptor (Radulovic et al, 1991; Preston et al, 1995), but it is undetectable in normal mucosa. One possible explanation is that the low level of expression of the GRP-R by the mucosa was not detectable above the background in the radioligand assays but was detectable in this study by the more sensitive RT-PCR. Only in those tumours with overexpression of GRP-R may there be sufficient binding for detection in radioligand binding assays. Alternatively, it may be that steady-state mRNA levels do not accurately reflect protein expression but, until validated receptor antibodies are available, we do not have the means to investigate this further. Our finding that GRP receptor mRNA is expressed in colonic epithelium is at variance with a recent report (Ferris et al, 1997). The absence of expression of NMB receptor mRNA demonstrated in this study is consistent with our studies using radioligand binding (Preston et al, 1995) where GRP receptor but not NMB receptor expression was demonstrated in tumour.

GRP mRNA and one of its spliced variants can be detected by RT-PCR in approximately equal proportions in all mucosal samples. As with the receptor, GRP ligand appears to be relatively overexpressed in tumour when compared to matched mucosa. The probe used for ISH studies detects mRNA both for GRP ligand and the spliced variant. Expression of both mRNA species is demonstrated in the mucosa rather than the stroma. In view of the lack of expression of the NMB-R in the tumour and mucosa, the finding of abundant NMB ligand in both was unexpected. As with the GRP-receptor and GPR, the expression of NMB was strongest in the tumours. Previous studies have provided evidence that GRP, NMB and other bombesin-like peptides can bind to either the GRP-receptor or NMB-receptor but differ in their relative affinities in different species (Jensen and Coy, 1991; Wang et al, 1992; Wang et al, 1993; Benya et al, 1995). At present we have little knowledge of the local concentrations of NMB and GRP at any

© 2000 Cancer Research Campaign one time, and therefore it is difficult to define their relative roles. It may be that NMB has paracrine activity on the adjacent smooth muscle (Bitar et al, 1993) providing a mechanism by which the mucosa may influence colonic motility.

In this small series it has not been possible to demonstrate a relationship between GRP and its receptor mRNA expression and conventional pathological features of colorectal cancer. It was observed, however, that more advanced tumours showed a lower level of expression. Confirmation of these finding would require a more accurately quantifiable method as well as a much larger series of samples.

This study provides further evidence for the presence of bombesin family ligands and receptors in the colon and rectum although their role in colorectal cancer remains to be determined. There have been several previous studies which support a role for the bombesin-like peptides in colorectal mitogenesis. Bombesin has been shown to stimulate rat colonic mucosal DNA synthesis (Johnson and Guthrie, 1983) and immunoreactive bombesin/GRP is abundant in the myenteric plexus and submucosal layer of the human colon (Price et al, 1984) where it may stimulate the growth of cancer cells in a paracrine manner. The murine colon cancer cell line MC-26 exhibits a dose dependent growth response to bombesin (Narayan et al, 1990). RC-3095, a bombesin antagonist, inhibits the growth of HT-29 human colon cancer xenografts in nude mice significantly reducing tumour volume, percentage change in tumour volume and tumour weights (Radulovic et al, 1991). Colo320HSR, a colon cancer cell line expresses GRP receptors and immunostains for GRP, suggesting an autocrine loop as seen in SCLC (own unpublished data). There are, however, other mechanisms by which bombesin-like peptides may stimulate cancer cell growth. GRP is known to affect the secretion of a number of gut peptides (Sunday et al, 1988) and may thus indirectly stimulate cell growth and division.

The findings in this study, together with the above, support a role for bombesin-like peptides in colorectal mitogenesis and tumorigenesis. They also support the potential of bombesin antagonists in the treatment of advanced colorectal cancer. Further progress in this area would benefit from the production of specific antibodies to the GRP receptor so that receptor expression could be correlated with prognosis in a large series of patients with colorectal cancer and a known outcome. Funded in part by the Wesser Medical Trust and the Wesser Cancer Trust.

### REFERENCES

Benya RV, Takashi K, Pradhan TK, Battey JF and Jensen RT (1995) Expression and characterization of cloned human bombesin receptors. *Mol Pharmacol* **47**: 10–20

Bitar KN and Zhu XX (1993) Expression of bombesin-receptor subtypes and their differential regulation of colonic smooth muscle contraction. *Gastroenterology* **105**: 1672–1680

Cancer Research Campaign (1993) *Cancer Research Campaign Factsheets 18.1–18.4.* Cancer Research Campaign: London

Cannon-Albright LA, Skolnick MH, Bishop DT, Lee RG and Burt RW (1988) Common inheritance of susceptibility to colonic adenomatous polyps and associated colorectal cancers. *New Engl J Med* **319**: 533–537

Corjay MH, Dobrzannski DJ, Way JM, Viallet J, Shapira H, Worland P, Sausville EA and Battey JF (1991) Two distinct bombesin receptor subtypes are expressed and functional in human lung cancer carcinoma cells. *J Biol Chem* **266**: 18771–18779

Cuttita F, Carney DN, Mulshine J, Moody TW, Fedorko J, Fischler A and Minna JD (1985) Bombesin-like peptides can function as autocrine growth factors in human small-cell lung cancer. *Nature* **316**: 823–826

Daly MC, Spindel ER, Giladi E, Grzeschik K and Naylor SL (1991) Proceedings, 11th Annual Int. Human Gene Mapping Meeting [Abstract]

Fathi Z, Corjay MH, Shapira H, Wada E, Benya R, Jensen R, Viallet J, Sausville EA and Battey JF (1993) BRS-3: a novel bombesin receptor subtype selectively expressed in testis and lung carcinoma cells. *J Biol Chem* **268**: 5979–5984

Ferris HA, Carroll RE, Lorimer DL and Benya RV (1997) Location and characterisation of the human GRP receptor expressed by gastrointestinal epithelial cells. *Peptides* **18**: 663–672

Groden J, Thliveris A, Samowitz W, Carlson M, Gelbert L, Albertson H, Stevens J, Spiro L and Robertson M (1991) Identification and characterisation of the familial adenomatous polyposis coli gene. *Cell* **66**: 589–600

Hirai M, Ishizuka J, Hirai A, Bold RJ, Townsend CM Jr and Thompson JC (1993) Bombesin stimulates intracellular $Ca^{2+}$ mobilization but not proliferation on human colon cancer cells. *Life Sci* **53**: 1859–1865

Jensen RT and Coy DH (1991) Progress in the development of potent bombesin receptor antagonists. *Trends Pharmacol Sci* **12**: 13–19

Johnson LR and Guthrie PD (1983) Regulation of antral gastrin content. *Am J Physiol* **245**: G725–G729

Krane IM, Naylor SL, Helin-Davis D, Chin WW and Spindel ER. Molecular cloning of cDNAs encoding the human bombesin-like peptide neuromedin B (1988) *J Biol Chem* **263**: 13317–13323

Leach FS, Nicolaides NC, Papadopoulos N, Liu B, Jen J, Parsons R, Pelyomäki P, Sistonen P, Aaltonen LA, Nyström-lahti M, Gua X-Y, Zhang J, Meltzer PS, Yu J-W, Kao F-T, Chen DJ, Cerosaletti KM, Fournier REK, Todd S, Lewis T, Leach RJ, Naylor SL, Weissenbach J, Mecklin J-P, Järvinen H, Petersen GM, Hamilton SR, Green J, Jass J, Warson P, Lynch HT, Trent JM, De La Chapelle A, Kinzler KW and Vogelstein B (1993) Mutations of a *mutS* homolog in hereditary nonpolyposis colorectal cancer. *Cell* **75**: 1215–1225

McDonald TJ, Jörnvall H, Nilsson G, Vagne M, Ghatei M, Bloom SR and Mutt V (1979) Characterization of a gastrin releasing peptide from porcine non-antral gastric tissue. *Biochem Biophys Res Commun* **90**: 227–233

Mantey SA, Weber HC, Sainz E, Akeson M, Ryan RR, Pradhan TK, Searles Rp, Spindel ER, Battey JF, Coy DH and Jensen RT (1997) Discovery of a high affinity radioligand for the human orphan receptor, bombesin receptor subtype 3, which

Minamino N, Kangawa K and Matsou H (1983) Neuromedin B: a novel bombesin-like peptide identified in porcine spinal cord. *Biochem Biophys Res Commun* **114**: 541–548

Narayan S, Guo Y-S, Townsend CM Jr and Singh P (1990) Specific binding and growth effects of bombesin-related peptides on mouse colon cancer cells in vitro. *Cancer Res* **50**: 6772–6778

Naylor SL, Sakaguchi AY, Spindel E and Chin WW (1987) Human gastrin-releasing peptide gene is located on chromosome 18. *Somat Cell Mol Genet* **13(1)**: 87–91

Preston SR, Ramsden CW, Miller GV and Primrose JN (1994) In vitro assay of bombesin and antagonists on human colon cancer cell line. *Br J Surg* **81**: 1819

Preston SR, Woodhouse LF, Jones-Blackett S, Miller GV and Primrose JN (1995) High affinity binding sites for gastrin releasing peptide on human colorectal cancer tissue but not uninvolved mucosa. *Br J Cancer* **71**: 1087–1089

Preston SR, Miller GV and Primrose JN (1996) Bombesin-like prptides and cancer. *Crit Rev Oncol Haematol* **23(3)**: 225–238

Price J, Penman E, Wass JAH and Rees LH (1984) Bombesin-like immunoreactivity in human gastrointestinal tract. *Regul Pept* **9**: 1–6

Radulovic SS, Miller G and Schally AV (1991) Inhibition of growth of HT-29 human colon cancer xenografts in nude mice by treatment with bombesin/gastrin releasing peptide antagonist (RC-3095). *Cancer Res* **51**: 6006–6009

Radulovic SS, Milanovanovic SR, Cai R-Z and Schally AV (1992) The binding of bombesin and somatostatin and their analogs to human colon cancers. *P.S.E.M.B.* **200**: 394–401

Rozengurt E and Sinnet-Smith J (1983) Bombesin stimulation of DNA synthesis and cell division in cultures of Swiss 3T3 cells. *Proc Natl Acad Sci USA* **80**: 2936–2940

Santen RJ, Manni A, Harvey H and Redmond C (1990) Endocrine treatment of breast cancer in women. *Endocrine Reviews* **11**: 221–265

Sausville EA, Lebacq-Verheyden AM, Spindel ER, Cuttuta F, Gazdar AF and Battey JF (1986) Expression of the gastrin-releasing peptide gene in human small cell lung cancer. Evidence for alternative precessing resulting in three distinct mRNAs. *J Biol Chem* **261**: 2451–2457

Schantz LJ, Naylor SL, Giladi E and Spindel ER (1991) Proceedings, 11th Annual Int. Human Gene Mapping Meeting. [Abstract]

Spindel ER, Chin WW, Price J, Rees LH, Besser GM and Habener JF (1984) Cloning and characterisation of cDNAs encoding human gastrin-releasing peptide. *Proc Natl Acad Sci USA* **81**: 5699–5703

Spindel ER, Zilderberg MD, Habener JF and Chin WW (1986) Two prohormones for gastrin-releasing peptide are encoded by two mRNAs differing by 19 bases. *Proc Natl Acad Sci USA* **83**: 19–23

© 2000 Cancer Research Campaign 

Spindel ER, Giladi E, Segerson TP and Nagalla S (1993) Bombesin-like peptides: of ligands and receptors. *Recent Prog Horm Res* **48**: 365–391

Sunday ME, Kaplan LM, Motoyama E, Chin WW and Spindel ER (1988) Gastrin releasing peptide (mammalian bombesin) gene expression in health and disease. *Lab Invest* **59**: 5–24

Vogelstein B, Fearon ER, Hamilton SR, Kern SE, Preisinger AC, Leppert M, Nakamura Y, White R, Smits AM and Bos JL (1988) Genetic alterations during colorectal-tumour development. *N Engl J Med* **319**: 525–532

Von Schrenck T, Heinz-Erain P, Moran T, Mantey SA, Gardener JD and Jensen RT (1989) Neuromedin B receptor in esophagus: evidence for receptor subtypes of bombesin receptors. *Am J Physiol* **256**: G747–G758

Wang LH, Battey JF, Wada E, Lin JT, Mantey S, Coy DH and Jensen RT (1992) Activation of neuromedin B-preferring bombesin receptors on rat glioblastoma $C_6$ cells increases cellular $Ca^{2+}$ and phosphoinositides. *Biochem J* **286**: 641–648

Wang LH, Mantey SA, Lin J-T, Frucht H and Jensen RT (1993) Ligand binding, internalization, degradation and regulation guanine nucleotides of bombesin receptor subtypes: a comparative study. *Biochim Biophys Acta* **1175**: 232–242

Wang QJ, Knezetic JA, Schally AV, Pour PM and Adrian TE (1996) Bombesin may stimulate proliferation of human pancreatic cancer cells through an autocrine pathway. *Int J Cancer* **68**: 528–534

Weber S, Zuckermann JE, Bostwick DG, Bensch KG, Sikic BI and Raffin TA (1985) Gastrin releasing peptide is a selective mitogen for small cell lung carcinoma in vitro. *J Clin Invest* **75**: 306–309

 *© 2000 Cancer Research Campaign*
